# Horizontal gene transfer after faecal microbiota transplantation in adolescents with obesity

**DOI:** 10.1186/s40168-024-01748-6

**Published:** 2024-02-12

**Authors:** Anna H. Behling, Brooke C. Wilson, Daniel Ho, Wayne S. Cutfield, Tommi Vatanen, Justin M. O’Sullivan

**Affiliations:** 1 Liggins Institute, University of Auckland, Auckland, New Zealand; 2 Institute of Biotechnology, Helsinki Institute of Life Science, University of Helsinki, Helsinki, Finland; 3 Research Program for Clinical and Molecular Metabolism, Faculty of Medicine, University of Helsinki, Helsinki, Finland; 4 The Broad Institute of MIT and Harvard, Cambridge, MA USA; 5 The Maurice Wilkins Centre, The University of Auckland, Private Bag 92019, Auckland, New Zealand; 6 Australian Parkinsons Mission, Garvan Institute of Medical Research, 384 Victoria Street, SydneyDarlinghurst, NSWNSW 2010 Australia; 7 MRC Lifecourse Epidemiology Unit, University of Southampton, Southampton, SO16 6YD UK; 8 Singapore Institute for Clinical Sciences, Agency for Science Technology and Research, Singapore, Singapore

**Keywords:** Horizontal gene transfer, Faecal microbiota transplantation, Human gut microbiome, Strain engraftment, Obesity

## Abstract

**Background:**

Horizontal gene transfer (HGT) describes the transmission of DNA outside of direct ancestral lineages. The process is best characterised within the bacterial kingdom and can enable the acquisition of genetic traits that support bacterial adaptation to novel niches. The adaptation of bacteria to novel niches has particular relevance for faecal microbiota transplantation (FMT), a therapeutic procedure which aims to resolve gut-related health conditions of individuals, through transplanted gut microbiota from healthy donors.

**Results:**

Three hundred eighty-one stool metagenomic samples from a placebo-controlled FMT trial for obese adolescents (the Gut Bugs Trial) were analysed for HGT, using two complementary methodologies. First, all putative HGT events, including historical HGT signatures, were quantified using the bioinformatics application WAAFLE. Second, metagenomic assembly and gene clustering were used to assess and quantify donor-specific genes transferred to recipients following the intervention. Both methodologies found no difference between the level of putative HGT events in the gut microbiomes of FMT and placebo recipients, post-intervention. HGT events facilitated by engrafted donor species in the FMT recipient gut at 6 weeks post-intervention were identified and characterised. Bacterial strains contributing to this subset of HGT events predominantly belonged to the phylum Bacteroidetes. Engraftment-dependent horizontally transferred genes were retained within recipient microbiomes at 12 and 26 weeks post-intervention.

**Conclusion:**

Our study suggests that novel microorganisms introduced into the recipient gut following FMT have no impact on the basal rate of HGT within the human gut microbiome. Analyses of further FMT studies are required to assess the generalisability of this conclusion across different FMT study designs and for the treatment of different gut-related conditions.

Video Abstract

**Supplementary Information:**

The online version contains supplementary material available at 10.1186/s40168-024-01748-6.

## Background

Horizontal gene transfer (HGT) is an agent of adaptive evolution enabling the transmission of DNA outside of direct ancestral lineages. HGT is best characterised within the bacterial kingdom and predominantly occurs through three mechanisms. Specifically, bacteria may (1) uptake free DNA from an environment (transformation), (2) transfer DNA directly between cells through pili (conjugation), or (3) acquire fragments of bacterial host DNA via bacteriophage infection (transduction) [1]. HGT also enables the transfer of mobile genetic elements encoding adaptive traits, including antibiotic resistance [2]. Consequently, HGT is of increasing public health interest because of its capacity to facilitate the dissemination of antibiotic-resistance genes across multiple, unrelated bacterial populations [3]. HGT is accentuated within biofilms that line the large intestinal mucosa [4]. HGT rates for human gut microbiomes are variable between individuals and are affected by the environment [5]. Notably, human gut microbiome HGT networks increase in complexity from birth to adulthood [6] and impact the transfer of antibiotic-resistance genes to both persistent and transient bacteria of the colon [7, 8]. However, relatively little is known about the patterns of HGT for non-antibiotic resistance genes within the human gut microbiome.

The increasing global prevalence of antibiotic-resistant infections has renewed attention to alternative approaches to conventional antibiotics [9]. Emerging evidence indicates that faecal microbiota transplantation (FMT) is a promising therapeutic approach for reducing the resistance gene load within an individual [10–12]. Patients undergoing FMT therapy are given a faecal transplant from clinically and microbiologically screened healthy donors, with the aim of restoring their gut microbiota from a state of disease. The therapy has already been adapted as a highly effective treatment for antibiotic-resistant recurrent *Clostridioides difficile* infection [13–16]. New paradigms that incorporate pivotal roles for the microbiome in complex phenotypes are leading to research into further treatment indications [17]. These include small trials that have demonstrated potential for treating obesity by FMT [18, 19]. During FMT, hundreds of microbial strains are transferred from the donor to the recipient [20]. The stable engraftment of donor strains into the microbiome of the recipient requires adaptation to the new environment and its resident microbial community. Theoretically, this adaptation may be supported by the horizontal transfer of genes with functions that are advantageous beyond a given niche [21, 22]. Therefore, we hypothesised that FMT treatment will increase HGT within the recipient’s gut microbiome.

HGT within the human gut microbiome can be characterised through a range of experimental and bioinformatic approaches [23]. Bioinformatic methodologies for analysing metagenomic data identify HGT through phylogenetic incongruencies or the altered composition of the DNA sequence [24]. Here, using previously published metagenome data from a randomised controlled trial investigating FMT for adolescent obesity [20], we characterise HGT events within participant and donor gut microbiomes. We apply two complementary methodologies to quantify HGT events across FMT and placebo recipients, capturing donor microbiome-specific genes transferred following the intervention, as well as all putative signatures of HGT in the metagenome through the inferred taxonomic discordance of genes on assembled metagenomic contigs [25].

## Methods

### Data acquisition

Quality-controlled, post-processed metagenomic sequencing files were obtained from a previously published clinical multi-donor FMT trial (the Gut Bugs Trial) [20]. The dataset included 381 samples from 87 recipients sampled at baseline, 6 weeks, 12 weeks, and 26 weeks post-treatment, and 9 donors sampled at each donation (Supplementary Table 1). Samples had an average post-QC read count of 45.7 million ± 6.1 million reads/sample (mean ± SD).

### Assembly of contig sequences

De novo contig assembly with MEGAHIT (version 1.1.4) [26] was used to produce contigs with a minimum length of 500 base pairs.

### Binning contigs to metagenome-assembled genomes

Genes were predicted from the contig sequences using Prodigal (version 2.6.3) [27]. Binning was subsequently performed using MetaBAT 2 (version 2.15–3) [28], to produce the metagenome-assembled genomes (MAGs). MAG completeness and contamination were assessed using CheckM (version 1.1.2) [29]. MAG taxonomy was assigned using GTDB-Tk (version 1.0.2, database release 89) [30]. To produce a non-redundant gene catalog, genes with > 95% identity were clustered using cd-hit-est (version 4.7) [31]. Functional annotations were generated using eggNOG (version 2.0.1) [32].

### Detection of horizontal gene transfer events

HGT events were identified with two complementary approaches. Firstly, to identify HGT events in the metagenomic sequencing data, contig sequences for each sample (*n* = 381) were analysed using WAAFLE (version 0.1.0) [25]. For each sample, WAAFLE produced output files with contigs that contained putative HGT events and those that did not contain any HGT events.

Secondly, we used a gene-based approach. Genes belonging to high-quality MAGs (thresholds of > 90% completeness and < 5% contamination) were included in this analysis. Across male and female sample data, 9,425,258 genes met the threshold criteria. 492,953 of these (5.23%) belonged to MAGs with no species classification and were subsequently excluded from HGT analyses. Male and female data were analysed separately given donor-recipient pairings were sex-matched [19]. Horizontally transferred gene clusters (HTGCs) were identified for each trial participant as gene clusters with discordant MAG species classification in the trial participant and donor samples. It was additionally required that any HTGCs were present in recipients at week 6 and in any respective donor samples, but absent from the trial participant at baseline. Data manipulation was performed in R (version 4.2.1) using the tidyverse package (version 1.3.2). Figures were produced using the R package ggplot2 (version 3.4.0), unless specified otherwise.

### Impact of FMT treatment on HGT events

The number of HGT events detected by WAAFLE in each of the 323 recipient samples was normalised by species richness data that was previously obtained using MetaPhlAn (version 2.7.7) [20]. All HGT events, including those without a direction of transfer, were used for this analysis. Linear mixed models were fitted (R package lme4, version 1.1.31; lmerTest version 3.1.3). Treatment group (placebo vs FMT), sex (female vs male), and timepoint (baseline vs week 6, week 12, and week 26) were considered fixed effects. Within-participant variation was considered a random effect. After comparing the fit of the full (interactions between the fixed effects) and reduced (no interactions between the fixed effects) models using a likelihood ratio test in the base R stats package (version 4.2.1) (*p* = 0.11), all two- and three-way interactions were excluded from the model. The 95% confidence intervals of the reduced model fixed effect coefficients were also obtained using the base R stats package.

HTGC frequency was calculated as the percentage of HTGCs relative to the total number of gene clusters for each sample. HTGC frequencies were compared between intervention groups, normality was assessed (Shapiro test; *p* = 1.93e − 07) and subsequently assessed by the Wilcoxon rank sum test. HTGCs that were unique to each treatment group were quantified. Distinct genes from the same gene cluster may originate from different samples and therefore were treated as different transfer events. The number of HGT events involving each HTGC was quantified for FMT- and placebo-specific HTGCs. Venn diagrams were produced using the R package Euler (version 7.0.0).

### Identification of strain engraftment-dependent horizontal gene transfer

Donor strain engraftment data was obtained from a previously published dataset [20]. This data was generated using StrainPhlan (MetaPhlAn, version 2.7.7) [33] which profiles the dominant strain for a given species within each sample. By comparing the genetic similarity of recipient strains pre- and post-intervention to the respective donor strains, instances of donor-strain engraftment were identified. To identify potential HGT events originating from donor-engrafting strains, HGT events were filtered based on instances of specific donor strain engraftment at 6 weeks post-FMT. Alluvial plots were produced using the R package ggalluvial (version 0.12.3).

### Taxonomic classification of horizontal gene transfer events

The engraftment-dependent HGT data for FMT recipients at week 6 was grouped by the engrafted donor and recipient bacterial species involved in the horizontal transfer, and the HGT events between each species combination were counted. Phylum-level identification for each species was performed. The species were grouped by phylum and plotted as a heatmap, with the color of each tile corresponding to the number of HGT events that occurred between each species combination.

The relative abundance of FMT donor bacterial phyla was calculated for the respective engrafted species and engrafted species with HGT. Engrafted phyla were defined as the phyla of donor bacterial strains that engrafted into the FMT recipient gut at week 6. This data was filtered for bacterial phyla only (i.e. no phage or archaea). Engrafted phyla with HGT were defined as engrafted phyla (see above) which were also identified as contributing to HGT in FMT recipients at week 6. Where the engrafted species were absent from MAG phylum classification, phylum classification was added manually using UniProt taxonomy [34]. Phylum relative abundances for the engrafted phyla and the engrafted phyla with HGT were compared using a chi-squared test from the base R stats package (version 4.2.1), with the Benjamini–Hochberg method used to control the false discovery rate for multiple hypothesis testing by adjusting the *p* values (R chisq.posthoc.test version 0.1.2). The distribution of phyla with donor-specific genes on high-quality MAGs (‘background’) was calculated as a reference.

### Retention of engraftment-dependent horizontally transferred genes

Gene abundances of each gene cluster were determined using the k-mer-based alignment tool KMA (version 1.2.27). The gene abundance data was subsequently subset for the engraftment-dependent HGT clusters in FMT recipients, post-intervention. Duplicate entries, i.e. multiple HGT events of the same gene cluster but involving different genes, were removed, as the abundance data corresponds to the abundance of the entire cluster. Linear mixed models with and without interactions between the fixed effects were fitted (R package lme4, version 1.1.31; lmerTest version 3.1.3) and all two- and three-way interactions were excluded from the model (likelihood ratio test, base R stats package, version 4.2.1; *p* = 0.6794). Sex (female vs male) and timepoint (week 6 vs week 12 and week 26) were considered fixed effects, while within recipient variation was considered a random effect. The 95% confidence intervals of the reduced model fixed effect coefficients were also obtained using the base R stats package.

### Functional annotation of engraftment-dependent horizontally transferred genes

Functional annotations of gene clusters were obtained using the eggNOG mapper (version 2.0.1) and included Cluster of Orthologous Genes (COG) categories. Functional categories were grouped by their major classification: cellular processes and signaling, information storage and processing, metabolism, or were poorly characterised. In cases where gene clusters had multiple functional categories assigned, these were considered separately. The relative abundances of HTGCs within each COG functional category classification were compared by a permutational multivariate analysis of variance (PERMANOVA) test with the Bray–Curtis method and 999 permutations, using the adonis2 function from the vegan R package (version 2.6.4).

## Results

### The Gut Bugs Trial

To understand the level of HGT in the gut microbiomes of FMT donors and recipients, we analysed metagenomic sequences obtained from a previously published double-blinded, randomised, placebo-controlled trial investigating the impact of FMT on adolescent obesity that was carried out in Auckland, New Zealand [18, 20]. Each recipient received capsules containing microbiota from four sex-matched donors, with the same donors used throughout the trial [19]. There was no effect of FMT on weight loss at 6 weeks post-intervention, although improvements in a marker of abdominal obesity (android-to-gynoid fat) were observed [18]. Post hoc analysis identified improvements in insulin sensitivity and glucose metabolism in a subset of participants with metabolic syndrome. Sustained shifts in both the structure and functional potential of recipient gut microbiomes in response to FMT were observed, with highly variable rates of strain engraftment [20].

### Metagenome-assembled genome (MAG) assembly

Metagenomic sequencing data were available for 381 trial samples, including 58 FMT donor samples collected from 9 donors over the 12-month stool donation period, and 323 samples belonging to 42 FMT and 45 placebo recipients collected at baseline and 6, 12, and 26 weeks post-intervention. An average of 96,604 contigs with a minimum length of 500 bp were assembled per sample (range 27,729–202,760). Clustering of genes with > 95% identity resulted in a gene catalog containing 2.9 million genes from across all samples. Across all samples, we assembled 20,941 metagenome-assembled genomes (MAGs), of which 4189 (20.0%) were high-quality (> 90% completeness and < 5% contamination). Most high-quality MAGs were taxonomically classified as belonging to the phyla Firmicutes (68.2%), Bacteroidetes (21.8%), and Actinobacteria (4.2%), and the species *Agathobacter rectale* (4.2%), *Agathobacter faecis* (4.1%), and *Faecalibacterium prausnitzii* (3.9%). A total of 9,425,258 genes were present on high-quality MAGs, representing 1,090,166 gene clusters, 79% of which were assigned a COG functional annotation.

### FMT does not increase HGT compared with a placebo

We analysed the assembled metagenomic contigs for historic HGT events by identifying DNA segments assigned to a separate ancestral lineage compared to the surrounding DNA [25]. We identified that 0.15% of assembled contigs per sample (range 0.074–0.26%) harbored segments implicated in HGT. The mean prevalence of contigs without HGT was 58.64% (range 38.19–80.33%) (Supplementary Fig. 1, Supplementary Table 2).

**Fig. 1:**
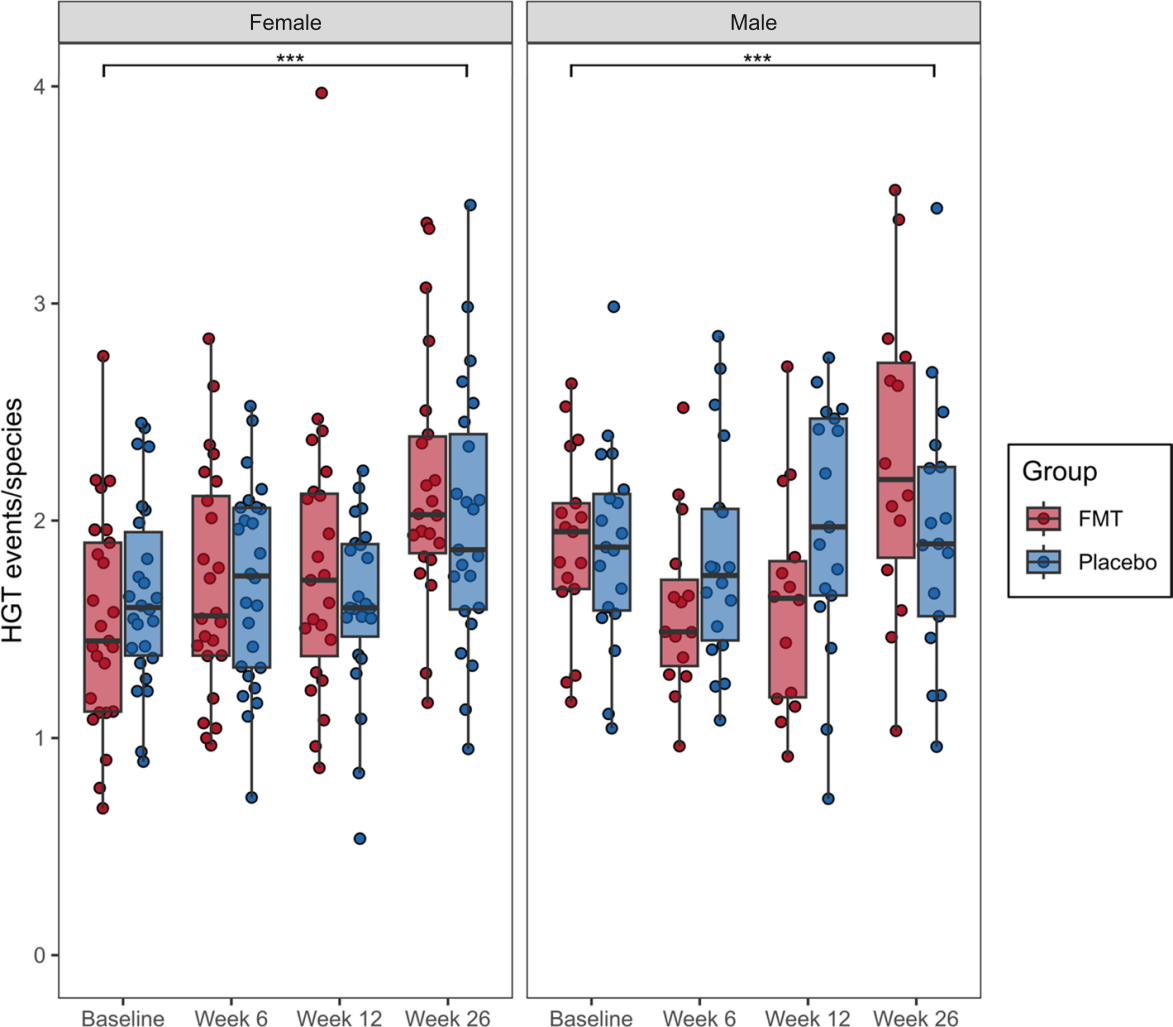
FMT treatment does not significantly increase HGT events in the recipient’s gut microbiome. HGT events were normalised by the number of microbial species present in each recipient sample (*n* = 323). Each point represents a participant’s sample. Boxes represent the interquartile range (IQR) split by the median, with whiskers expanding up to 1.5 × the IQR. Differences between groups over time were assessed using linear mixed models (group, sex, and timepoint fixed effects and recipient random effect). There were no significant differences between treatment groups, but a significant difference between baseline and week 26 was observed for both groups, ***p < 0.001. HGT, horizontal gene transfer; FMT, faecal microbiota transplantation

We hypothesised that the acute competition associated with FMT would stress the recipient microbiome and lead to an increase in HGT events. However, it is also possible that metagenome samples with increased microbial species richness have an increased propensity for HGT events. Therefore, we normalised HGT event counts within each microbiome sample by species richness. Linear mixed models did not identify evidence of a treatment effect on normalised HGT events (LMM, *b* = 0.0076, 95%CI [− 0.11, 0.12], *p* = 0.90). Similarly, there was no evidence of a significant sex (*b* = 0.11, 95%CI [− 0.0036, 0.23], *p* = 0.060), or timepoint effect from baseline to week 6 (*b* =  − 0.0043, 95%CI [− 0.16, 0.15], *p* = 0.96), or baseline to week 12 (*b* = 0.032, 95%CI [− 0.13, 0.19], *p* = 0.71). By contrast, there was a significant longitudinal effect, in both FMT and placebo recipients, from baseline to week 26 (*b* = 0.37, 95%CI [0.21, 0.53], *p* < 0.001) (Fig. 1), which is likely due to drift over time.

**Fig. 2:**
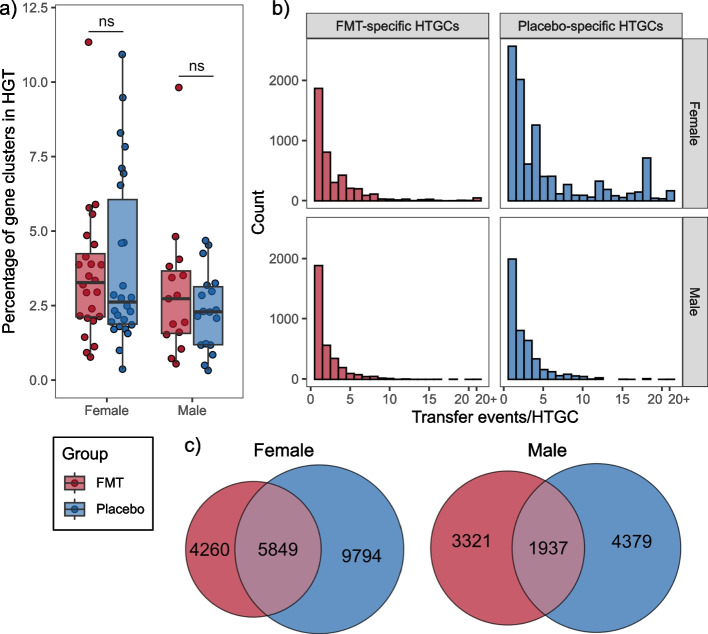
FMT does not increase HGT rates following treatment, when compared with a placebo. **a** The percentage of HTGCs was calculated from the number of distinct recipient gene clusters involved in HGT for each FMT (*n* = 39) or placebo (*n* = 44) recipient relative to the number of distinct gene clusters on high-quality MAGs for each respective participant, at week 6. The percentage of HTGCs was compared between groups (Wilcoxon test). Each point represents a participant’s sample. Boxes represent the interquartile range (IQR) split by the median, with whiskers expanding up to 1.5 × the IQR. **b** The number of transfer events for each HTGC was plotted separately for the FMT-specific and placebo-specific HTGCs, for males and females. A bin width of 1 was used. HTGCs with greater than 20 transfer events were grouped under ‘20 + ’. **c** Counts of FMT-specific (red), shared (purple), and placebo-specific (blue) HTGCs in females and males. HGT, horizontal gene transfer; FMT, faecal microbiota transplantation; HTGC, horizontally transferred gene cluster; MAG, metagenome assembled genome; ns, not-significant (*p* < 0.05)

A limitation of the aforementioned approach to detect HGT events is its inability to differentiate between HGT events that have occurred following FMT treatment and historical signatures of HGT present in bacterial genomes. We hypothesised that HGT due to FMT treatment, specifically, would increase following FMT. Therefore, we utilised a complementary method to quantify the number of FMT donor genes that transferred to FMT recipients, at 6 weeks post-intervention. Genes that were present on high-quality MAGs (i.e. > 90% completion and < 5% contamination) were clustered using a > 95% identity threshold. To identify putative HGT events, we performed a time-course analysis for each individual (FMT and placebo recipients). Gene clusters that were present within the participant samples at 6 weeks post-intervention, absent at baseline, and were also present in the respective donor samples were identified. Gene clusters were then classified as being horizontally transferred if their taxonomic identification differed between donor and recipient MAGs. Using this approach we identified 57,590 putative HGT events occurring post-intervention in 39 FMT recipients. Using the same criteria, we observed 111,273 putative HGT events that occurred in 44 placebo recipients. Adjusting for gene richness, there was no difference in the percentage of genes involved in HGT for the FMT and placebo groups, including when subset by sex (Wilcoxon rank sum test, overall, *p* = 0.56; males, *p* = 0.84; females, *p* = 0.63). This finding is consistent with our earlier observations and supports the conclusion that FMT does not increase the rate of HGT events above the background rates in the gut microbiome (Fig. 2a).

To understand the occurrence of treatment-specific HGT events, we quantified the number of transfer events for each horizontally transferred gene cluster (HTGC), across FMT- and placebo-specific HTGCs. FMT-specific HTGCs were defined as distinct HTGCs that were present in FMT recipients at 6 weeks post-intervention and absent in the respective placebo group samples. Placebo-specific HTGCs were defined as the reverse. We observed that 42% (4260/10,109) of HTGCs in female FMT recipients were FMT-specific and 63% (9794/15,643) of HTGCs in female placebo recipients were placebo-specific. In males, 63% (3321/5258) of HTGCs in FMT recipients were FMT-specific and 69% (4379/6316) of HTGCs in placebo recipients were placebo-specific (Fig. 2c). Quantifying the number of individual transfer events for each FMT-specific and placebo-specific HTGC identified that FMT- and placebo-specific HTGCs were predominantly involved in single transfer events (FMT-specific mean 2.7 ± 3.2; placebo-specific mean 4.7 ± 5.4). Therefore, we did not observe any increase in the occurrence of FMT-specific HGT events, relative to the background rate of HGT observed in the placebo cohort (Fig. 2b).

### Engrafted bacterial species horizontally transfer genes to recipient bacteria

**Fig. 3:**
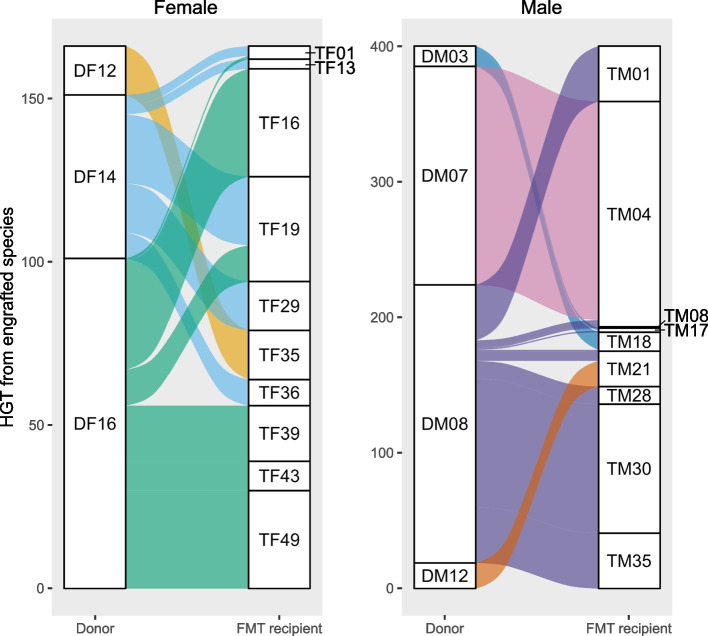
Engrafted bacterial strains from FMT donors transfer genes to the recipient’s bacteria. Alluvial plots illustrating HGT events where gene clusters from engrafted donor species were transferred to distinct bacterial species within the FMT recipient (6 weeks post-FMT). Alluvial flow is coloured according to FMT donor identity. HGT, horizontal gene transfer; FMT, faecal microbiota transplantation

The engraftment of microbial strains within the recipient microbiomes was observed to be donor-specific in the Gut Bugs FMT trial for adolescent obesity [20]. We hypothesised that the engraftment of novel donor strains into the recipient’s gut would promote HGT with other microorganisms within the recipient’s gut. To investigate this, we selectively focused on HGT events that occurred at 6 weeks post-intervention between donor-engrafting strains and distinct strains in the FMT recipient’s gut [20]. HGT events involving engrafted bacteria were identified for three female donors and four male donors. Engrafted strains contributing to HGT events in FMT recipients most commonly originated from donor DF16 in females and donor DM08 in males (Fig. 3), consistent with the higher levels of strain engraftment observed from these donors [20]. Between the male donor DM07 and recipient TM04, specifically, there were a high number (*n* = 161) of HGT events, with 158 of these being between *Bacteroides uniformis* and *Bacteroides vulgatus*.

**Fig. 4:**
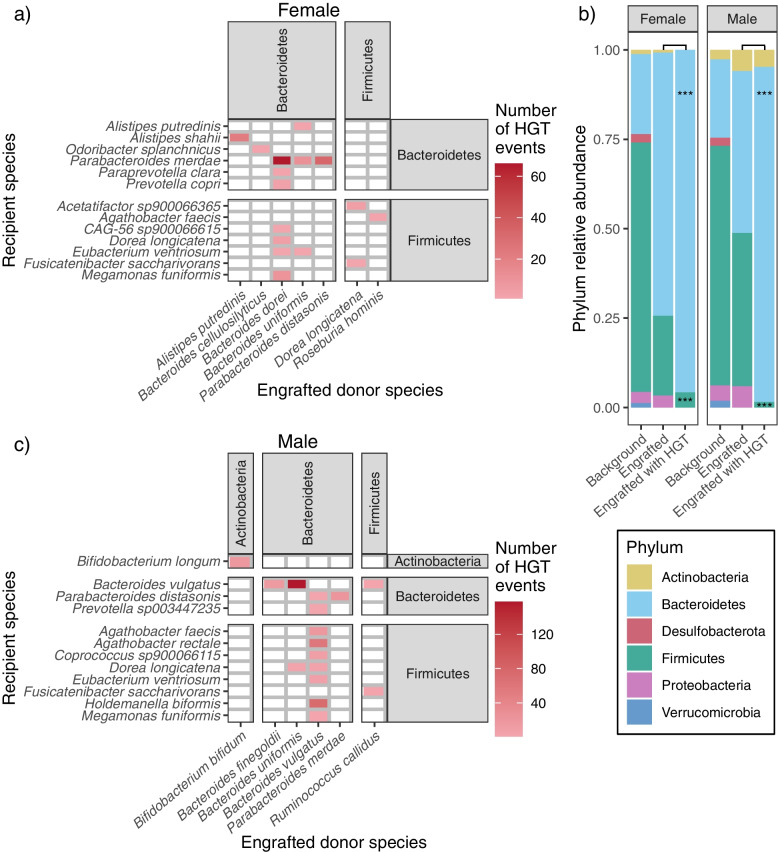
Bacteroidetes are overrepresented contributors to HGT. HGT events between donor-engrafted species and recipient species in **a** females and **c** males, clustered by phylum. Tiles are coloured by the number of HGT events between each donor-recipient species pairing. **b** Relative abundances of bacterial phyla across donor genes on high-quality MAGs (‘background’), donor bacterial phyla engrafted in FMT recipient gut microbiomes at week 6 (‘engrafted’), and those engrafted strains that contributed to HGT (‘engrafted with HGT’). Phylum distributions for engrafted and engrafted with HGT bacteria were compared (chi-squared test). ***p < 0.001. HGT, horizontal gene transfer; FMT, faecal microbiota transplantation; MAG, metagenome assembled genome

We investigated if the HGT events associated with engrafted bacteria occurred through inter- or intra-phylum transfers. The majority of HGT events were facilitated by engrafted species in the Bacteroidetes phylum (159/166 events for females and 375/400 events for males) (Figs. 4a and c). We compared the distribution of engrafted phyla that contributed to HGT, with all engrafted phyla, to determine if the Bacteroidetes phylum was overrepresented amongst the engrafted donor bacteria (Fig. 4b). In the female cohort, Bacteroidetes were overrepresented (adj. *p* < 0.001, chi-squared test) and Firmicutes were underrepresented (adj. *p* < 0.001, chi-squared test) in the engrafted bacteria contributing to HGT. In the male cohort, Bacteroidetes were overrepresented (adj. *p* < 0.001, chi-squared test), while Firmicutes and Proteobacteria were underrepresented (both adj. *p* < 0.001, chi-squared test) in the engrafted bacteria contributing to HGT.

### Engraftment-dependent HTGCs are maintained following FMT

**Fig. 5:**
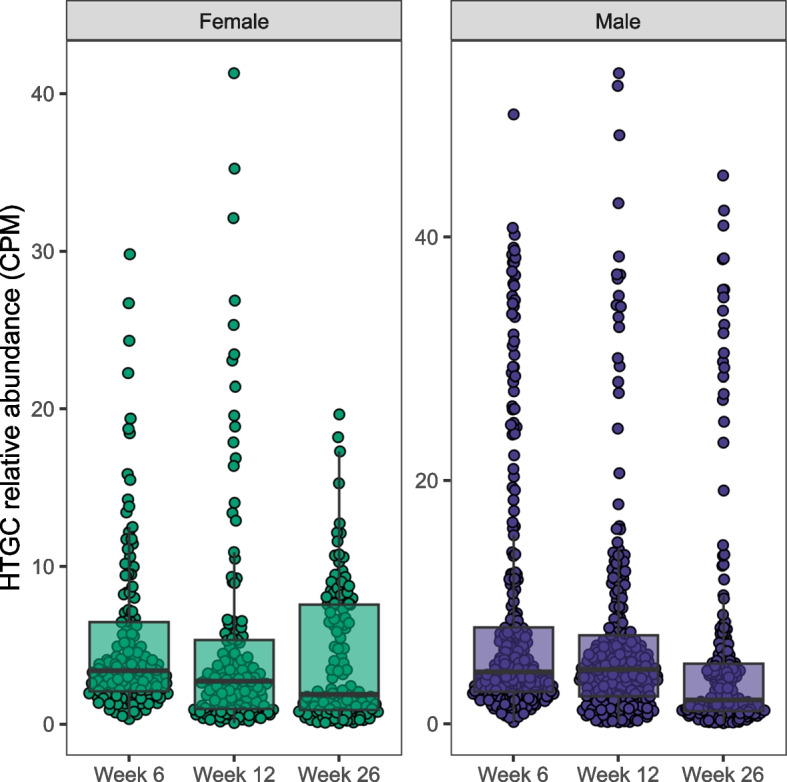
Engraftment-dependent HTGCs are maintained in the FMT recipient’s gut. Relative abundance (CPM) of HTGCs transferred from engrafted FMT donor strains to FMT recipient bacteria. Each point represents a distinct HTGC. Boxes represent the interquartile range (IQR) split by the median, with whiskers expanding up to 1.5 × the IQR. Abundance data was measured at 6-, 12-, and 26 weeks post-FMT and compared using linear mixed models (sex and timepoint fixed effects and recipient random effect). HTGC, horizontally transferred gene cluster; CPM, copies per million reads

We assessed the retention of engraftment-dependent HTGCs in FMT recipients. Gene abundance data was obtained for each engraftment-dependent HTGC in the FMT recipients, post-intervention. We hypothesised that HTGCs acquired by 6 weeks post-intervention would further proliferate within participants at week 12 and week 26. In total, there were 289 male and 139 female distinct engraftment-dependent HGT events detected in FMT recipients, 6 weeks following the intervention. Of these, 248 (85.8%) male and 134 (96.4%) female HTGCs were retained at week 12, while 232 (80.3%) and 135 (97.1%) female HTGCs were retained at week 26. We found no evidence of timepoint- or sex-specific effects on the relative abundance of engraftment-dependent HTGCs using linear mixed models to compare the relative abundance of HTGCs at 6-, 12-, and 26 weeks post-intervention (Fig. 5). These data suggest that while the HTGCs did not proliferate, they were maintained within the host for up to 26 weeks following the intervention.

### Functional annotation of engraftment-dependent HTGCs

**Fig. 6:**
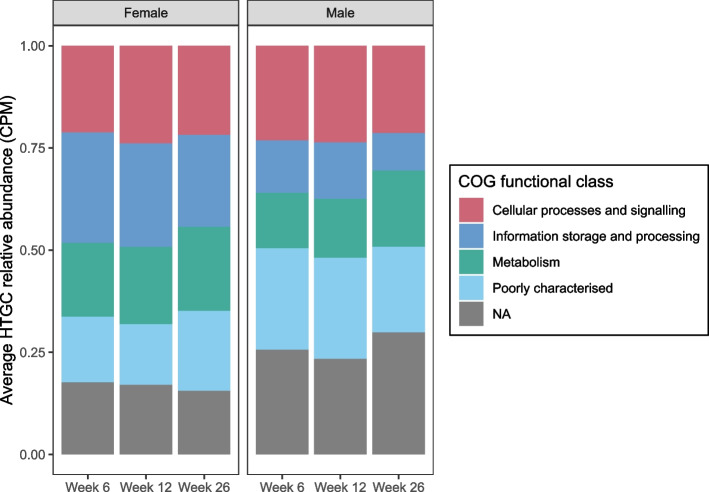
Gene functions of engraftment-dependent HTGCs show no differences up to 26 weeks post-intervention. COG functional categories were grouped by classification. Relative abundances of gene cluster classifications were compared within each sex at each timepoint (PERMANOVA test). CPM, copies per million reads; COG, clusters of orthologous groups; HTGC, horizontally transferred gene cluster

We investigated the assigned COG functions for the engraftment-dependent HTGCs [35]. Across the female recipients, 76 (54.7%) of engraftment-dependent HTGCs detected at 6 weeks post-intervention were able to be assigned a functional classification (cellular processes and signalling, information storage and processing, or metabolism), while 37 (26.6%) were poorly characterised, and 26 (18.7%) had no classification. In the male cohort, 166/289 (57.4%) had a functional classification, 62/289 (21.5%) were poorly classified, and 61/289 (21.1%) had no classification (Fig. 6). Comparisons of the relative abundance of gene clusters with each functional classification at each timepoint identified no significant differences (PERMANOVA test females, *p* = 0.87; males, *p* = 0.92). The relative abundance of individual COG functional categories assigned to these HTGCs was also not significant (PERMANOVA test females, *p* = 0.92; males, *p* = 0.99) (Supplementary Fig. 2).

## Discussion

We investigated HGT dynamics within the human gut following FMT using metagenomic data from a placebo-controlled FMT trial for obesity in adolescents [18, 20]. We compared the overall rates of gene transfer in the gut microbiomes of the participants and the impact of FMT on this process. We observed that the rate of HGT did not significantly differ between participants in the FMT and placebo cohorts. However, engrafted bacterial strains originating from the FMT donors did facilitate HGT, and these transferred genes were maintained within the recipient microbiomes up to 26 weeks post-intervention, suggesting that HGT is relevant to the long-term modulation of the human gut microbiome.

Infant gut microbiomes obtain mobile genetic elements from maternal gut microbial strains [36], although this overlap can vary considerably between mother-infant pairings [37]. Maternal bacteria of the order Bacteroidales have been identified as key contributors to these transfer events [36], which is consistent with the overrepresentation of engrafted Bacteroidetes facilitating HGT following FMT observed in our study. Similarly, a global study of HGT in human gut microbiomes identified Bacteroides species as being more promiscuous for HGT than species from other phyla [5]. Notably, within the global populations that were analysed, Groussin and colleagues identified higher rates of HGT in the gut microbiomes of industrialised populations, which may be due to lifestyle-related factors [5].

HGT could be facilitated by the presence of transient species within the recipient’s gut. However, the transfer of genes between species is limited by species proximity [38] and therefore more likely to be facilitated by engrafted species. Our data shows that engrafted donor bacteria facilitated HGT with recipient bacteria. It is possible that HGT is an important mechanism for the FMT donor microbiome to modulate the functions of the recipient gut microbiome community. Consequently, the influence of the FMT donor microbiome on the recipient may not necessarily depend on prolonged strain engraftment, if genes from the engrafted microorganism are transferred before the species themselves are lost. We were unable to determine how long an engraftment is required to facilitate HGT and propose that this should be empirically measured.

We acknowledge a number of potential limitations associated with our study. First, the analyses we performed were constrained by the structure of the FMT clinical trial [19]. For example, the timepoints for which metagenomic data were available limited the periods over which HGT could be analysed. It is possible that there was an enhanced rate of transient HGT closer to the FMT itself, and data from timepoints before 6 weeks post-intervention would have identified a greater differential in HGT rates between the FMT and placebo recipients. However, as this was not observed at 6 weeks post-intervention, any potential dissimilarity prior to that point must have been transient (i.e. happening in bacteria that were lost from the recipient’s microbiome). Alternatively, differences in HGT levels in response to FMT may not have been observed due to the high basal rates of HGT in the human gut, particularly in an urban cohort [5]. It is also possible that we did not detect any statistical changes due to the limited sample size. The Gut Bugs Trial comprised 87 participants that produced 323 samples, which is higher than many other clinical FMT trials [39], while a recent study investigating HGT in the human gut microbiome used a cohort of 48 individuals [5]. This highlights the importance of larger-scale studies, in general, for assessing the impact of FMT on the gut microbiome.

Quorum sensing may play a role in HGT [40, 41], requiring a certain population of bacteria to trigger the widespread transfer of genes between species. Therefore, the bowel cleanse administered prior to the FMT intervention in this particular study may have prevented the bacteria from reaching the required threshold, leaving a microbial density that was too low for HGT events to occur. The method we used to identify genes that were transferred from FMT donors to the recipients was equally as successful at identifying putative HTGCs in the placebo cohort. Given the participants of the placebo cohort were not treated with donor FMT, they must have acquired HTGCs after the intervention that, by chance, were also present within the respective (male/female) donor cohort. Therefore, it is not possible to definitively assign the HTGCs acquired by the FMT cohort as originating from the respective donor microbiomes, without also considering the possibility that these too were acquired by chance from the environment.

Accurate taxonomic assignment of genes is fundamental to HGT identification. The possible misassignment of species during MAG binning could have under- or overestimated the rate of HGT, in a species-dependent manner. For example, it is possible that a high number of transfer events we observed between the male donor DM07 *Bacteroides uniformis* and recipient TM04 *Bacteroides vulgatus* may have been due to a misclassification of the bacterial taxonomy; however, it is also not possible to prove this from our data. It remains possible that the use of MAG binning approaches in the identification of HTGCs may also lead to an underestimation of the HGT rate. Binning tools, including MetaBAT 2, have demonstrated low rates of plasmid recovery [42]. Therefore, our findings have likely only captured transferred genes which integrated into the chromosome. The use of a complementary approach, such as Hi-C [43], to capture plasmid transfer may provide a more comprehensive understanding of the rate of HGT (i.e. including both episomal and chromosomal events) following FMT treatment.

Despite these limitations, the finding that there is no impact of FMT on HGT rates in the human gut microbiome is notable. Moreover, our study has the strength of combining two complementary approaches to quantify the rate of HGT in metagenomic data. These methodologies are transferable to the analysis of other FMT trials. We suggest that their application to additional FMT studies may further elucidate the impact of FMT on HGT rates in human gut microbiomes.

## Conclusions

In conclusion, FMT does not increase rates of HGT in human subjects. However, donated strains do participate in HGT and evidence for these events is retained for at least 6 months following the FMT treatment itself. The observation of similar rates and apparently identical events in placebo patients indicates that the rates of HGT are not limited by access to donor strains and the gut microbiome is subject to regular genetic influx from the wider environment. Future analyses of other FMT trials will reveal the extent to which HGT patterns following FMT are conserved across different conditions and treatment methodologies.

### Supplementary Information

**Additional file 1: Table S1.** Number of participants in each group at each timepoint.

**Additional file 2: Table S2.** Number of contigs with horizontal gene transfer (HGT) per sample from the WAAFLE output.

**Additional file 3: Supplementary Fig. 1.** Less than one percent of metagenomic contig sequences from healthy individuals and obese adolescents contain evidence of HGT. The percentage of contigs with and without HGT is plotted for each of the 381 microbiome samples from the Gut Bugs Trial [20]. ‘Unclassified’ contigs could not be explained by a single species or species-pair. HGT, horizontal gene transfer. **Supplementary Fig. 2.** Gene functions of engraftment-dependent HTGCs show no differences up to 26 weeks post-intervention. Relative abundances of gene clusters in each COG functional category were compared within each sex at each timepoint (PERMANOVA test). COG functional category descriptions: [C] Energy production and conversion; [D] Cell cycle control, cell division, chromosome partitioning; [E] Amino acid transport and metabolism; [F] Nucleotide transport and metabolism; [G] Carbohydrate transport and metabolism; [H] Coenzyme transport and metabolism; [I] Lipid transport and metabolism; [J] Translation, ribosomal structure and biogenesis; [K] Transcription; [L] Replication, recombination and repair; [M] Cell wall/membrane/envelope biogenesis; [N] Cell motility; [O] Posttranslational modification, protein turnover, chaperones; [P] Inorganic ion transport and metabolism; [Q] Secondary metabolites biosynthesis, transport and catabolism; [S] Function unknown; [T] Signal transduction mechanisms; [U] Intracellular trafficking, secretion, and vesicular transport; [V] Defense mechanisms. CPM, copies per million reads; COG, clusters of orthologous groups; HTGC, horizontally transferred gene cluster.

## Data Availability

The metagenomic dataset analysed during the current study is available in the NCBI SRA repository (BioProject PRJNA637785 https://www.ncbi.nlm.nih.gov/bioproject/PRJNA637785/). Additional data files are available on request. Data processing R scripts are available at https://github.com/annabehling/gutbugs_hgt/.

## Abbreviations

COG: Clusters of orthologous groups
CPM: Copies per million
FMT: Faecal microbiota transplantation
HGT: Horizontal gene transfer
HTGC: Horizontally transferred gene cluster
MAG: Metagenome assembled genome
PERMANOVA: Permutational multivariate analysis of variance
