# Supplementary material for: Horizontal gene transfer after faecal microbiota transplantation in adolescents with obesity
Source: Microbiome. 2024 Feb 12;12:26. doi: 10.1186/s40168-024-01748-6 (PMC10860221; doi:10.1186/s40168-024-01748-6)
Supplement: Supplementary file 4 — Additional file 3: Supplementary Fig. 1. Less than one percent of metagenomic contig sequences from healthy individuals and obese adolescents contain evidence of HGT. The percentage of contigs with and without HGT is plotted for each of the 381 microbiome samples from the Gut Bugs Trial [20]. ‘Unclassified’ contigs could not be explained by a single species or species-pair. HGT, horizontal gene transfer. Supplementary Fig. 2. Gene functions of engraftment-dependent HTGCs show no differences up to 26 weeks post-intervention. Relative abundances of gene clusters in each COG functional category were compared within each sex at each timepoint (PERMANOVA test). COG functional category descriptions: [C] Energy production and conversion; [D] Cell cycle control, cell division, chromosome partitioning; [E] Amino acid transport and metabolism; [F] Nucleotide transport and metabolism; [G] Carbohydrate transport and metabolism; [H] Coenzyme transport and metabolism; [I] Lipid transport and metabolism; [J] Translation, ribosomal structure and biogenesis; [K] Transcription; [L] Replication, recombination and repair; [M] Cell wall/membrane/envelope biogenesis; [N] Cell motility; [O] Posttranslational modification, protein turnover, chaperones; [P] Inorganic ion transport and metabolism; [Q] Secondary metabolites biosynthesis, transport and catabolism; [S] Function unknown; [T] Signal transduction mechanisms; [U] Intracellular trafficking, secretion, and vesicular transport; [V] Defense mechanisms. CPM, copies per million reads; COG, clusters of orthologous groups; HTGC, horizontally transferred gene cluster. [file 40168_2024_1748_MOESM3_ESM.pdf]

Supplementary Figures

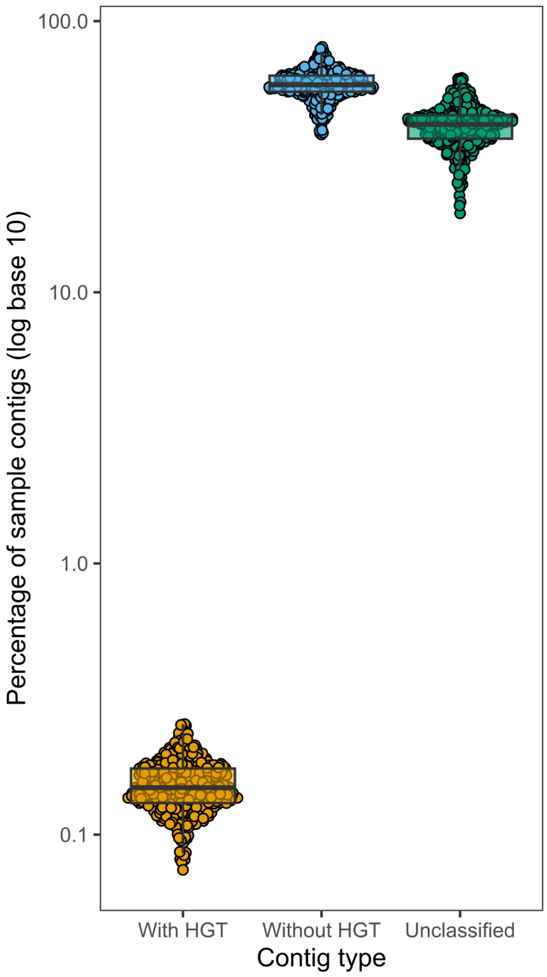

Supplementary Figure 1. Less than one percent of metagenomic contig sequences from healthy individuals and obese adolescents contain evidence of HGT. The percentage of contigs with and without HGT is plotted for each of the 381 microbiome samples from the Gut Bugs Trial (Wilson et al. 2021). 'Unclassified' contigs could not be explained by a single species or species-pair. HGT, horizontal gene transfer.

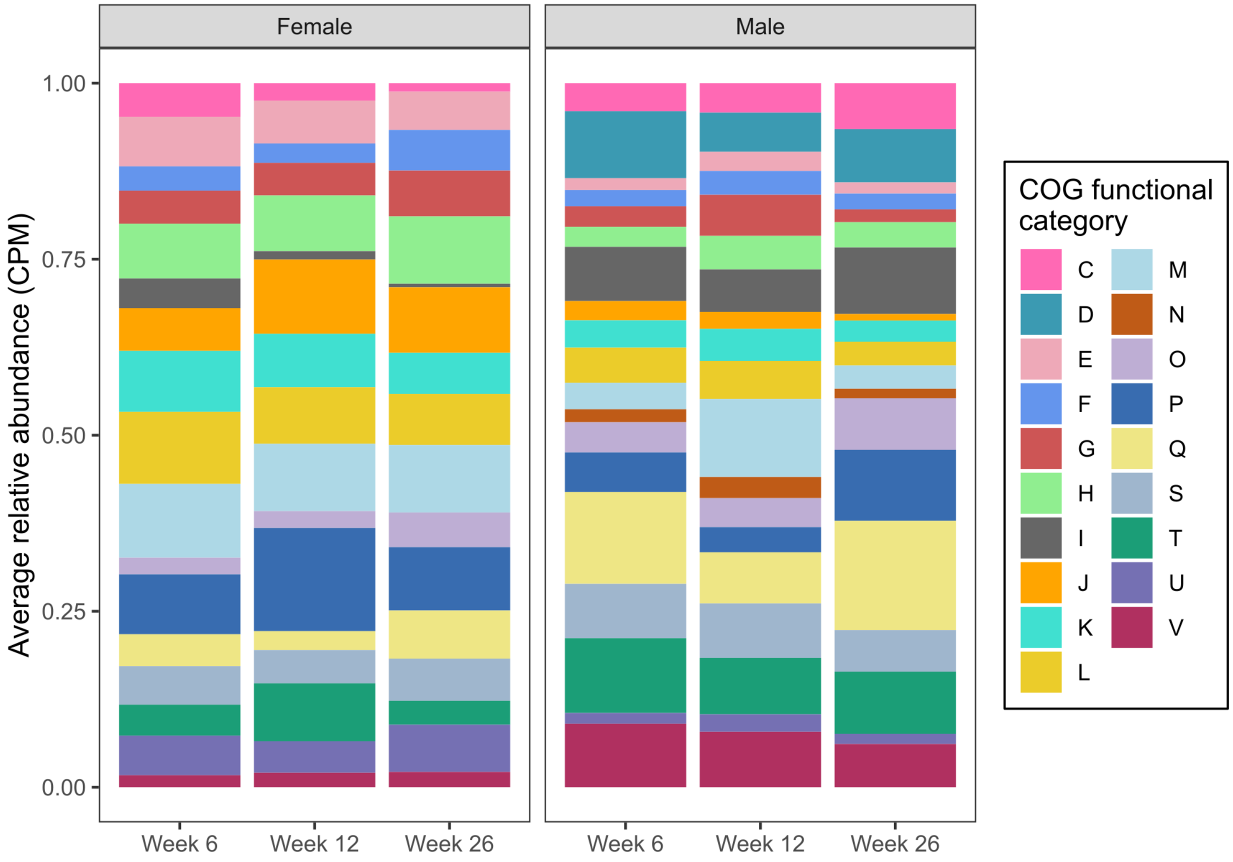

Supplementary Figure 2. Gene functions of engraftment-dependent HTGCs show no differences up to 26 weeks post-intervention. Relative abundances of gene clusters in each COG functional category were compared within each sex at each timepoint (PERMANOVA test). COG functional category descriptions: [C] Energy production and conversion; [D] Cell cycle control, cell division, chromosome partitioning; [E] Amino acid transport and metabolism; [F] Nucleotide transport and metabolism; [G] Carbohydrate transport and metabolism; [H] Coenzyme transport and metabolism; [I] Lipid transport and metabolism; [J] Translation, ribosomal structure and biogenesis; [K] Transcription; [L] Replication, recombination and repair; [M] Cell wall/membrane/envelope biogenesis; [N] Cell motility; [O] Posttranslational modification, protein turnover, chaperones; [P] Inorganic ion transport and metabolism; [Q] Secondary metabolites biosynthesis, transport and catabolism; [S] Function unknown; [T] Signal transduction mechanisms; [U] Intracellular trafficking, secretion, and vesicular transport; [V] Defense mechanisms. CPM, copies per million reads; COG, clusters of orthologous groups; HTGC, horizontally transferred gene cluster.
